# Recent Advances in Understanding and Engineering Polyketide Synthesis

**DOI:** 10.12688/f1000research.7326.1

**Published:** 2016-02-23

**Authors:** Wenjun Zhang, Joyce Liu

**Affiliations:** 1 Department of Chemical and Biomolecular Engineering, University of California, Berkeley, CA, 94720, USA; 2 Department of Bioengineering, University of California, Berkeley, CA, 94720, USA

**Keywords:** Polyketides, polyketide synthesis, polyketide synthase

## Abstract

Polyketides are a diverse group of natural products that form the basis of many important drugs. The engineering of the polyketide synthase (PKS) enzymes responsible for the formation of these compounds has long been considered to have great potential for producing new bioactive molecules. Recent advances in this field have contributed to the understanding of this powerful and complex enzymatic machinery, particularly with regard to domain activity and engineering, unique building block formation and incorporation, and programming rules and limitations. New developments in tools for *in vitro* biochemical analysis, full-length megasynthase structural studies, and *in vivo* heterologous expression will continue to improve our fundamental understanding of polyketide synthesis as well as our ability to engineer the production of polyketides.

## Introduction

**Figure 1.**
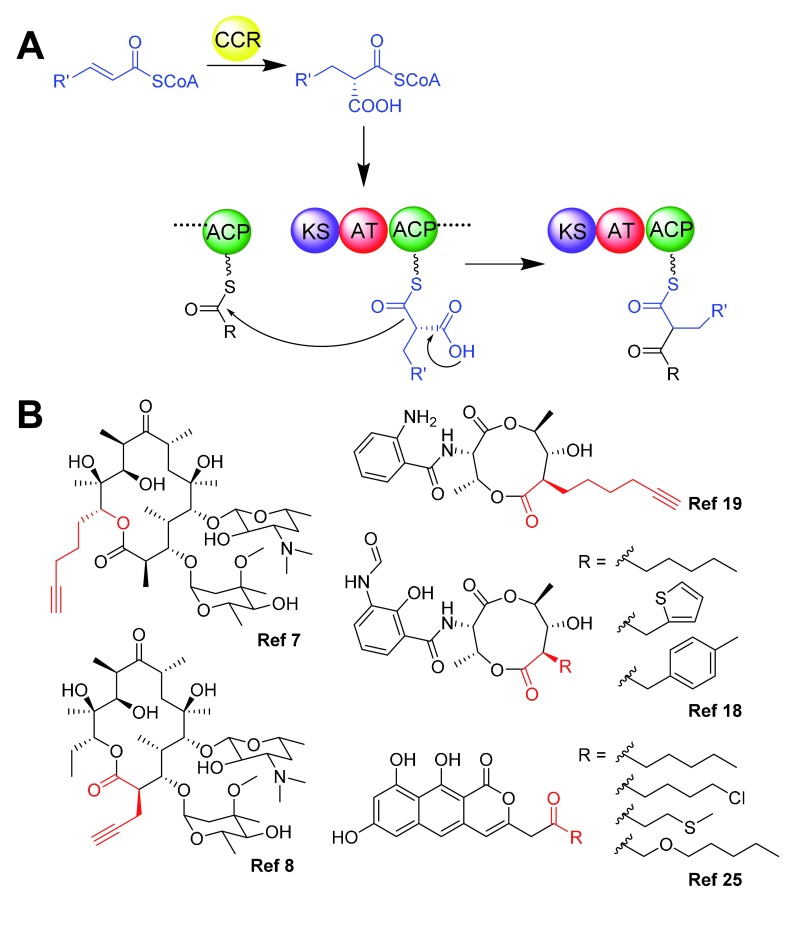
Novel polyketides generated by the incorporation of atypical building blocks. ( **A**) Scheme showing crotonyl-CoA carboxylase/reductase (CCR)-catalyzed biosynthesis of unusual extender units and their subsequent incorporation into polyketide scaffolds. Possible R’ groups include aliphatics, alkyl halides, and aromatics; R represents the donor polyketide intermediate. ( **B**) Structures of novel polyketides with the modifications from atypical building blocks shown in red.

Polyketide compounds are a large family of natural products with great structural diversity and complexity. Many of these compounds are valued for their potent biological activities, and particularly well-known examples include erythromycin, tetracycline, rifamycin, and lovastatin. Polyketides are formed by a family of enzymes known as polyketide synthases (PKSs), which often operate in an assembly line-like fashion to join together acyl coenzyme A (CoA) building blocks ^1^. The core catalytic domains of type I and II PKSs include the ketosynthase (KS) domain, which is responsible for catalyzing decarboxylative Claisen condensations for chain extension; the acyltransferase (AT) domain, which is responsible for building block selection and loading; and the acyl carrier protein (ACP) domain, on which the polyketide chain is elongated ( Figure 1A). Additional enzymes that may be either part of the PKS megasynthase or standalone can modify the nascent polyketide chain during or post assembly, and these enzymes further contribute to the diversity and complexity of polyketides that can be produced.

Because of the inherent modularity of many PKSs and their vast potential for producing pharmaceutically relevant compounds, there has been longstanding interest in engineering these enzymes to produce novel polyketides in a predictable manner. Decades of work ranging from the characterization of PKSs with unique attributes to the fundamental understanding of how these enzymes function to the manipulation of catalytic domains and modules to generate unnatural products have brought us closer to this goal ( Figure 1B) ^2, 3^. In this short commentary, we discuss recent advances in these areas, focusing on both the new knowledge about PKSs that has been garnered and the tools that have been developed to facilitate efforts in PKS engineering. While the body of work on PKSs is vast, we have restricted our discussion here to a few selected themes found in PKS research from the past couple of years.

## Recent advances in understanding polyketide synthesis

As the “gatekeeping” domain, the AT domain has been a key target for polyketide engineering, and a number of recent studies continue to focus on understanding and engineering building block selection by this domain, particularly ATs that recognize atypical substrates, i.e. substrates other than malonyl-CoA or methylmalonyl-CoA. For example, an AT domain from the monensin PKS ^4^ and the loading AT from the avermectin PKS ^5^, both with relaxed substrate specificities, were subjected to computational modeling and structural analysis, respectively, to enable the identification of residues responsible for substrate binding and specificity. Similarly, the AT domain of ZmaA, which specifically recognizes the precursor hydroxymalonyl-ACP, was investigated through structural and biochemical analysis, and this study showed that the donor carrier protein itself biased extender unit selection ^6^. Based on the promiscuity of the loading AT and a mutation in one of the extending ATs of 6-deoxyerythronolide B synthase (DEBS), short-chain alkynoic building blocks have also been incorporated into the backbone of erythromycin ( Figure 1B) ^7, 8^. In addition, several other recent studies have centered on *trans*-ATs, which may be easier to engineer than ATs acting in *cis*. Through *in vitro* kinetic studies, it was shown that although *trans*-ATs can complement inactivated *cis*-ATs, which can be a useful approach for introducing alternative extender units into polyketides, the efficiency of *trans*-AT complementation is greatly affected by the identity of both the carboxyacyl-CoA and the ACP substrates ^9^. Promisingly, several key electrostatic interactions that define the interaction epitope between a *trans*-AT promiscuous to carboxyacyl-CoA substrates and its cognate ACP were identified, and this knowledge was further leveraged to engineer a noncognate ACP into a detectable substrate for this *trans*-AT via the introduction of a single amino acid substitution ^10^. In addition, a *trans*-AT has also been used to site-selectively incorporate fluorine into a polyketide backbone ^11^.

Aside from these AT domain studies, there have also been studies focused on the biosynthetic aspect of unique PKS building blocks. In particular, promiscuous malonyl-CoA synthetase variants have been used to synthesize a broad range of malonyl-CoA extender units substituted at the C2 position ^12, 13^. In addition, a family of crotonyl-CoA carboxylase/reductase (CCR) enzymes that transform α,β-unsaturated acyl-CoA substrates to the corresponding carboxyacyl-CoA extender units have also received great attention. CCR enzymes are typically quite flexible and have already been demonstrated to generate both aliphatic CoA- and aromatic CoA-linked extender units ( Figure 1A) ^14– 17^. The recent structure-based engineering of a CCR enzyme from antimycin biosynthesis afforded the production of several new polyketide extender units ^18^. This work also demonstrated the first use of heterocyclic and substituted arene extender units by PKS machinery ( Figure 1B), though it is notable that not all of the generated α-substituted malonyl-CoAs could be accepted by the PKS. In addition to CoA building blocks, dedicated ACP-dependent pathways are often found to generate atypical building blocks for PKSs as well. One example is our recently elucidated ACP-dependent terminal alkyne biosynthetic machinery, which was further exploited for the *in situ* generation and incorporation of terminal alkynes into polyketide scaffolds as alkynoic starter or extender units ( Figure 1B) ^19^. This work demonstrates the feasibility of *de novo* biosynthesis of terminal alkyne-tagged polyketides that can be subjected to *in situ* biorthogonal chemistry for further modification. Another recent example is the DH*-KR* (dehydratase-ketoreductase) bifunctional proteins that were characterized to convert glyceryl- *S*-ACP into the unusual lactyl- *S*-ACP starter unit in FR901464 and lankacidin biosynthesis ^20^.

Another area of focus in recent PKS understanding and engineering lies in iterative type I PKSs, which are typically found in fungi, though more examples are now emerging from bacteria. Although numerous bioactive polyketides are synthesized by iterative type I PKSs, unlike the well-known linear assembly line of multi-modular type I PKSs, these fungal PKSs have only one module that is used iteratively, and the related programming rules regarding substrate selection, catalytic domain utilization in each elongation cycle, regiospecific modification, polyketide chain length control, chain release and transfer, *etc.* are only just beginning to be understood ^21, 22^. For the relatively simple class of non-reducing iterative type I PKSs (NR-PKSs) that are involved in aromatic polyketide synthesis, the starter unit ACP transacylase (SAT) domain has been the focus of several recent studies including domain swapping to generate new aromatic polyketides ^23^ and structural analysis to identify the basis for acyl unit selection ^24^, since unnatural starter units are often properly processed by the rest of the catalytic domains of NR-PKSs ( Figure 1B) ^25^. In addition, systematic *in vitro* domain swapping of NR-PKSs followed by examination of the resulting on-target and shunt products highlighted the important effects of chain length control by KS domains, editing by thioesterase (TE) domains, and inter-domain interactions on combinatorial biosynthesis ^26^. While some additional work has also been done on highly and partially reducing PKSs ^27, 28^, particularly with regards to combinatorialization with NR-PKSs ^29^ or nonribosomal peptide synthetases (NRPSs) ^30^, the relative lack of understanding about the programming of these types of PKSs makes them more difficult to engineer effectively.

## Recent developments in tools for understanding and engineering PKSs

As polyketide engineering continues to progress, the development of tools and strategies to understand the underlying PKS programming rules and exploit them for the diversification and overproduction of new polyketide compounds is of paramount importance. In this vein, *in vitro* biochemical analysis using purified enzymes continues to serve as the most important method for studying PKS enzymology, particularly in understanding the precise function and substrate specificity of catalytic domains, reaction mechanisms, and the internal kinetics of the catalytic program. Despite recent significant improvements in the sensitivity and accuracy of mass spectrometry ^31^, it remains challenging to directly detect and quantify the majority of ACP-bound biosynthetic intermediates of polyketides. Alternatively, total enzymatic reconstitution of polyketide synthesis *in vitro* serves as one of the most common assay methods for biochemical analysis ^16, 32, 33^. Additionally, several unique quantitative and facile assay strategies have been developed recently; of notable interest are those coupling PKS-catalyzed reactions and fluorescent click chemistry ^10, 34^. In addition to biochemical assays, structural analysis continues to play a vital role in studying PKSs, paving the way for a more detailed understanding of the mechanism and dynamics of PKSs, particularly with the advent of structural knowledge of full-length megasynthases. New strategies have recently been developed to address the technical challenges in obtaining structural information for large PKS protein complexes. For example, small-angle X-ray scattering (SAXS) analyses of megasynthases combined with the rigid-body refinement of the high-resolution domain structures have been used to model modular structures of DEBS, leading to the proposal of a disc-shaped module that can cage the ACP at the center of a ring formed by the other PKS domains ^35^. This method can be used to probe the solution state and obtain structural information for catalytically active megasynthases, and it is a powerful approach for modeling large, dynamic macromolecular complexes. In parallel, electron cryo-microscropy has also recently been used to determine the reconstruction of a full-length PKS module and showed that a single reaction chamber provides the intramodular ACP with access to all of the catalytic sites while the ACP from the upstream module uses a separate entrance to deliver the polyketide intermediate ^36^. An accompanying study further examined this PKS in different catalytic states, providing new insight into the structural rearrangements involved in chain elongation ^37^. Thus, electron cryo-microscopy is emerging as a powerful tool for studying complex PKSs by enabling high-resolution information about the overall structure, organization, and dynamics of complete PKS modules to be obtained directly.

In addition to *in vitro* analysis, *in vivo* study of PKSs, particularly heterologous expression of PKSs and their auxiliary enzymes, has proven to be invaluable for the understanding and production of polyketides, as reflected by the recent developments in new heterologous expression tools and the successes in polyketide production by a wide range of heterologous hosts. While phage-mediated homologous recombination such as λ Red/ET recombineering has long been used for the direct capture of gene clusters from bacterial artificial chromosomes and genomic DNA ^38^, transformation-associated recombination (TAR)-based techniques that typically rely on homologous recombination in yeast and allow for the capture of much larger clusters are quickly gaining traction ^3, 39– 42^. The development of a *Saccharomyces cerevisiae*− *Escherichia coli* shuttle−actinobacterial chromosome integrative capture vector for use with TAR further demonstrates the relative ease and speed with which gene clusters can now be heterologously expressed ^43^. The selection of the heterologous host also remains an important consideration for polyketide production, and popular choices of hosts currently include *E. coli*, *S. cerevisiae*, *Streptomyces*, and *Aspergillus*, which are all continually being engineered to promote higher compound titers ^3, 39, 44^. In general, it is best to use a host similar to the native one, as differences in regulation, codon usage, and biosynthetic precursors may result in difficulties with heterologous expression. Nonetheless, the complete refactoring of gene clusters may be used to achieve heterologous expression in hosts that differ significantly from the native producers, and efforts to refactor gene clusters by adding promoters or deleting regulatory elements as well as to engineer the metabolism of heterologous hosts for increased product titers are now easier than ever with tools like CRISPR/Cas, TAR, and λ Red/ET recombineering ^3, 39, 43, 45– 48^.

## Concluding remarks

Looking to the future, an improved understanding of the intriguing PKS machinery remains critical for the successful engineering of polyketide synthesis, and biochemical, in particular quantitative and mechanistic, analyses as well as structural studies of these megasynthases will continue to play important roles in revealing the underlying PKS programming rules. Meanwhile, this research field will continue to benefit from advances in research toolkits, especially new bioinformatics, synthetic biology, and analytical tools, all of which can help lead to the production of new polyketide compounds for drug discovery and development.

## Abbreviations

PKS, polyketide synthase; CoA, coenzyme A; KS, ketosynthase; AT, acyltransferase; ACP, acyl carrier protein; DEBS, 6-deoxyerythronolide synthase; CCR, crotonyl-CoA carboxylase/reductase; DH, dehydratase; KR, ketoreductase, NR-PKS, non-reducing polyketide synthase; SAT, starter unit ACP transacylase; TE, thioesterase; NRPS, nonribosomal peptide synthetase; SAXS, small angle X-ray scattering; TAR, transformation-associated recombination.
